# A clinically interpretable machine learning model for early detection of diabetic retinopathy in multiple community health centers

**DOI:** 10.3389/fendo.2026.1834629

**Published:** 2026-05-04

**Authors:** Juncheng Tong, Aifa Tang, Lifang Liu, Luyuan Zhang, Hainan Wang, Mengyuan Qu, Bing Liu

**Affiliations:** 1 Department of Basic Medical Sciences, School of Medicine, Shenzhen University, Shenzhen, China; 2 The Third Affiliated Hospital of Shenzhen University, Shenzhen Luohu People’s Hospital, Shenzhen, China

**Keywords:** clinical prediction model, diabetic retinopathy, machine learning, primary care, risk stratification

## Abstract

**Background:**

Early detection of diabetic retinopathy (DR) remains challenging in primary care, where access to ophthalmic screening is limited. We developed and validated a prediction model using routinely collected health data to identify diabetic patients at increased risk of DR.

**Methods:**

This retrospective study included 1,475 diabetic patients from three community health centers in China. The cohort was split into a development set (n = 1,177) and a held-out test set (n = 298). We developed three machine learning models using 5-fold cross-validation: penalized logistic regression (GLMNET), extreme gradient boosting (XGBoost), and random forest (Ranger). Model performance was evaluated using the area under the receiver operating characteristic curve (AUROC), area under the precision-recall curve (AUPRC), Brier score, calibration, and decision curve analysis. Feature importance was assessed using SHapley Additive exPlanations (SHAP).

**Results:**

DR prevalence was 13.5%. In the test set, GLMNET achieved an AUROC of 0.770 (95% CI 0.671–0.856) and an AUPRC of 0.452 (95% CI 0.325–0.620). Its Brier score was 0.095, with a calibration intercept of 0.206 and a calibration slope of 0.953. XGBoost showed comparable discrimination, whereas Ranger performed less favorably. Decision curve analysis suggested possible net benefit across threshold probabilities from 10% to 40%. SHAP analyses identified urine glucose as the most influential predictor.

**Conclusions:**

This model showed moderate discrimination and acceptable but imperfect calibration in a three-center community-based cohort. Its use of routinely collected variables and transparent model structure suggests potential value for risk stratification in primary care, but external validation and prospective implementation studies are required before routine clinical use.

## Introduction

Diabetic retinopathy (DR) is one of the most prevalent microvascular complications of diabetes mellitus and a primary cause of preventable blindness in the global working-age population (1, 2). According to recent data from the International Diabetes Federation, DR affects nearly one-quarter of the estimated 500 million diabetic individuals worldwide (3). Regular fundus screening is widely recommended, and early detection can prevent up to 95% of severe visual impairment (4). However, the implementation of universal ophthalmic screening in primary care settings faces strong obstacles. In low- and middle-income countries, adherence to ophthalmology referrals remains dismally low—ranging from 13% to 17% (5). These gaps are particularly evident in community health centers where specialized equipment and ophthalmologists are limited.

Existing approaches to DR risk stratification have evolved considerably in recent years. Traditional prediction models have demonstrated the feasibility of using clinical variables—including type and duration of diabetes, HbA1c, and blood pressure—to identify individuals at high risk (6, 7). Recently, large-scale studies from multiple cohorts have identified more predictors such as age, glycemic control, renal function markers, and medication use (8, 9). However, these studies also highlight some limitations: many existing models are derived from hospital-based cohorts with established disease, limiting their applicability to asymptomatic populations in primary care; moreover, studies often overemphasize discriminative performance while neglecting model calibration and clinical utility across decision thresholds (10). A recent systematic review of DR prediction models concluded that while published models showed decent performance, the majority were at high risk of bias due to inadequate validation and poor transparency (11).

Machine learning offers promising avenues to address certain limitations by capturing complex nonlinear relationships among multiple predictors. Studies have shown that ensemble methods such as XGBoost and random forest can achieve superior discrimination compared to conventional logistic regression in some contexts (12, 13). However, the trade-off between predictive performance and interpretability remains a major concern for clinical adoption. “Black-box” models, despite strong statistical metrics, face challenges in real-world workflows where clinicians require transparency and trust. Furthermore, most machine learning studies have focused on tertiary care populations, with limited evaluation in community-based settings where the majority of diabetes management occurs (14).

To address these gaps, we developed and validated a DR prediction model using routinely collected health data from three community health centers in China. Our study population reflects real-world primary care—including not only conventional high-risk diabetic patients but also younger individuals and those in early stages of health management. We prioritized model interpretability and predictive performance. We aimed to provide a clinically actionable tool for early risk stratification in resource-limited primary care settings.

## Methods

### Study design and participants

This retrospective study included patients with diabetes from three community health centers (Cuizhu, Guiyuan, and Liantang) in Shenzhen, China, between January 2018 and December 2025. Eligible participants were aged ≥18 years, had type 1 or type 2 diabetes mellitus diagnosed according to World Health Organization criteria, and underwent at least one fundus examination during the study period. Baseline demographic, laboratory, and urinalysis variables were extracted from routinely collected clinical records. Patients were excluded if they had ocular diseases other than diabetic retinopathy (DR) that could affect retinal assessment, previous ocular surgery or retinal laser treatment, pregnancy or lactation, severe systemic disease with limited life expectancy, or unavailable outcome data.

A total of 1,682 records were screened, and 1,475 participants were included in the final analysis. The dataset was randomly divided into a development set (80%, n=1,177) and an independent test set (20%, n=298) using stratified sampling according to DR status.

### Outcome definition

The primary outcome for model development was the presence of DR, defined as a binary outcome (DR vs no DR). DR status was ascertained from routine fundus examinations using standardized clinical assessment across centers. For the purposes of this study, any stage of DR was considered a positive outcome. Because the analytic dataset used for model development contained a binary DR indicator only and did not include a structured severity variable, severity-stratified modeling was not feasible.

### Predictors and data preprocessing

Candidate predictors were selected from routinely available baseline clinical variables, including demographic characteristics, anthropometric measurements, blood pressure, hematologic indices, glycemic markers, liver and renal function parameters, lipid profiles, urinalysis variables, and study center information. The full predictor list is provided in the **Supplementary Material**.

Data preprocessing was performed using the development set only. Missing numeric variables were imputed by the median, categorical variables were encoded as appropriate, and continuous variables were standardized. The same preprocessing parameters were then applied to the test set. Variable-wise missingness in the overall cohort, development set, and test set before imputation is summarized in **Supplementary Table S1**.

### Model development and validation

Three models were developed: penalized logistic regression (GLMNET), gradient boosting (XGBoost), and random forest (Ranger). Model hyperparameters were optimized by 5-fold cross-validation. After tuning, each model was refitted on the full development set and evaluated in the independent test set.

Of the three models, GLMNET was chosen as the primary model because it provided the best overall balance of discrimination, calibration, parsimony, and interpretability.

### Model performance assessment

Model performance was evaluated in the independent test set. Discrimination was quantified by the area under the receiver operating characteristic curve (AUROC) and the area under the precision-recall curve (AUPRC). Overall predictive performance was assessed with the Brier score. Calibration was examined using calibration plots, together with the calibration intercept and slope. Clinical utility was evaluated by decision curve analysis.

Differences in AUROC between models were compared with the DeLong test, whereas differences in AUPRC were assessed using a bootstrap-based method. For the final GLMNET model, performance at prespecified probability thresholds was additionally described by sensitivity, specificity, positive predictive value, negative predictive value, accuracy, F1 score, balanced accuracy, and positive and negative likelihood ratios. Exploratory center-stratified analyses, urine-glucose sensitivity analysis, and sensitivity analyses related to class-imbalance handling were also conducted for the final model and are reported in the **Supplementary Material**.

### Model interpretability analysis

We applied SHAP (SHapley Additive exPlanations) to interpret the final GLMNET model. SHAP values quantify the contribution of each predictor to individual risk estimates. For each patient in the test set, we computed SHAP values and visualized them using summary plots to display global feature importance and the direction of each predictor’s effect. This analysis provides insight into how specific clinical variables influence the model’s predictions, supporting clinical interpretability.

### Statistical analysis

Continuous variables are presented as median (interquartile range) or mean ± standard deviation, as appropriate, and categorical variables as number (percentage). Baseline characteristics were summarized for the development and test sets, as well as for participants with and without DR. All analyses were performed in R software (version 4.5.2). A two-sided P value <0.05 was considered statistically significant.

## Results

### Cohort characteristics

**Figure 1:**
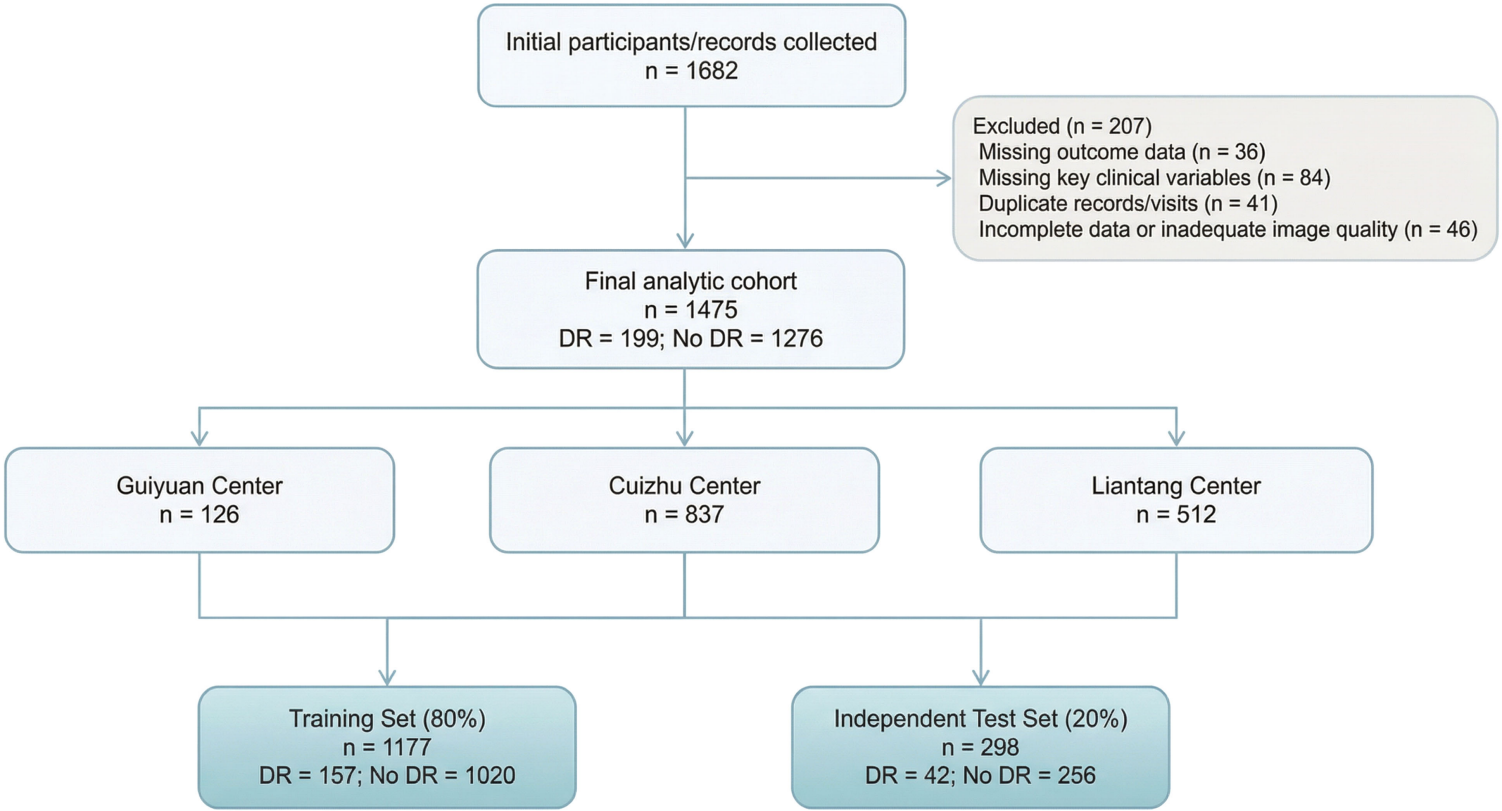
Patient inclusion flowchart. Flow diagram of participant screening, exclusions, and final cohort assembly for the DR prediction study.

Among the 1,682 patients initially screened, 1,475 participants were ultimately included in the analysis after excluding ineligible cases (**Figure 1**). The overall prevalence of diabetic retinopathy (DR) was 13.5% (199 of 1,475). Following a predefined 8:2 ratio, 1,177 participants were assigned to the development set and 298 to the independent test set. A supplementary figure summarizing the final analytic cohort and the predefined fixed train–test split is provided in **Supplementary Figure S1**.

**Table 1** presents the baseline characteristics. The median age was 66.8 years (IQR 59.6–71.7), with a balanced sex distribution (49.4% male). Participants were predominantly from the Cuizhu center (56.7%), followed by Liantang (34.7%) and Guiyuan (8.5%). Glycemic control was generally adequate, as reflected by median fasting plasma glucose of 5.2 mmol/L (IQR 4.8–6.1) and HbA1c of 5.9% (IQR 5.5–6.4). Lipid profiles and renal function markers were within expected ranges for a community-based diabetic population. Baseline characteristics were comparable between the development and test sets, with no meaningful differences in center distribution, DR status, or other clinical variables. Variable-wise missingness before imputation is summarized in **Supplementary Table S1**.

**Table 1:** Baseline characteristics of the overall cohort and the development and test sets.

Study population	Overall	Development set	Test set	P value	SMD
N	1475	1177	298		
Center, n (%)				0.986	0.011
Cuizhu	837 (56.7)	669 (56.8)	168 (56.4)		
Guiyuan	126 (8.5)	100 (8.5)	26 (8.7)		
Liantang	512 (34.7)	408 (34.7)	104 (34.9)		
DR status, n (%)				0.733	0.022
No DR	1276 (86.5)	1020 (86.7)	256 (85.9)		
DR	199 (13.5)	157 (13.3)	42 (14.1)		
Demographic characteristics					
Age, years	66.84 (59.60, 71.72)	66.80 (59.50, 71.54)	67.00 (60.09, 72.03)	0.506	0.048
Sex, n (%)				0.117	0.102
Male	728 (49.4)	593 (50.4)	135 (45.3)		
Female	747 (50.6)	584 (49.6)	163 (54.7)		
Weight, kg	63.80 (56.50, 72.40)	63.80 (56.40, 72.20)	63.80 (56.73, 72.62)	0.837	0.017
WC, cm	85 (79, 91)	85 (79, 91)	85 (79, 92)	0.596	0.050
BMI, kg/m²	24.46 (22.43, 26.79)	24.42 (22.44, 26.69)	24.76 (22.30, 27.05)	0.278	0.074
Blood pressure					
SBP, mmHg	127 (117, 139)	127 (117, 139)	127 (116, 138)	0.879	0.011
DBP, mmHg	81(74, 87)	81 (74, 87)	81 (73, 87)	0.955	0.020
Glycemic variables					
FPG, mmol/L	5.2 (4.8, 6.1)	5.2 (4.8, 6.1)	5.2 (4.8, 6.0)	0.572	0.021
HbA1c, %	5.9 (5.5, 6.4)	5.9 (5.5, 6.4)	5.8 (5.5, 6.4)	0.564	0.023
Hematologic variables					
Hb, g/L	139 (130, 150)	140 (130, 150)	138 (129, 151)	0.359	0.068
WBC, ×10^9^/L	6.02 (5.06, 7.09)	6.02 (5.06, 7.08)	6.03 (5.15, 7.09)	0.788	0.009
PLT, ×10^9^/L	226 (193, 265)	226 (192, 263)	228 (196, 272)	0.179	0.136
Liver function					
ALT, U/L	18 (14, 26)	18(14, 26)	19 (14, 26)	0.825	0.056
AST, U/L	20 (18, 24)	20 (18, 24)	21 (17, 24)	0.981	0.004
TBil, μmol/L	12.4 (10.0, 15.5)	12.3 (10.0, 15.5)	12.6 (10.0, 15.7)	0.604	0.046
DBil, μmol/L	4.0 (3.0, 5.3)	4.0 (3.0, 5.3)	4.1 (3.1, 5.3)	0.562	0.034
IBil, μmol/L	8.4 (6.7, 10.4)	8.4 (6.7, 10.3)	8.4 (6.7, 10.5)	0.539	0.039
Renal function					
Scr, μmol/L	69 (59, 82)	69 (59, 83)	68 (58, 80)	0.313	0.024
BUN, mmol/L	5.3 (4.4, 6.2)	5.3 (4.4, 6.2)	5.4 (4.4, 6.2)	0.856	0.059
UAlb, mg/L	13.10 (6.70, 26.85)	13.00 (6.60, 27.30)	14.10 (7.28, 25.62)	0.681	0.074
Lipid profile mmol/L					
TC	4.73 (4.04, 5.47)	4.71 (4.04, 5.46)	4.85 (4.07, 5.51)	0.224	0.071
TG	1.35 (0.98, 1.97)	1.34 (0.97, 1.96)	1.39 (1.00, 2.03)	0.383	0.080
LDL-C	2.85 (2.20, 3.43)	2.83 (2.17, 3.41)	2.91 (2.26, 3.53)	0.089	0.117
HDL-C	1.23 (1.04, 1.49)	1.23 (1.03, 1.49)	1.23 (1.07, 1.45)	0.516	0.022
Urinalysis					
Urine protein, n (%)				0.898	0.088
Negative	1202 (81.5)	955 (81.1)	247 (82.9)		
Trace	123 (8.3)	99 (8.4)	24 (8.1)		
1+	94 (6.4)	76 (6.5)	18 (6.0)		
2+	31 (2.1)	27 (2.3)	4 (1.3)		
3+	17 (1.2)	13 (1.1)	4 (1.3)		
Missing	8 (0.5)	7 (0.6)	1 (0.3)		
Urine glucose, n (%)				0.709	0.135
Negative	1258 (85.3)	996 (84.6)	262 (87.9)		
Trace	12 (0.8)	11 (0.9)	1 (0.3)		
1+	22 (1.5)	18 (1.5)	4 (1.3)		
2+	12 (0.8)	9 (0.8)	3 (1.0)		
3+	53 (3.6)	46 (3.9)	7 (2.3)		
4+	110 (7.5)	90 (7.6)	20 (6.7)		
Missing	8 (0.5)	7 (0.6)	1 (0.3)		
Urine ketone, n (%)				0.757	0.096
Negative	1411 (95.7)	1122 (95.3)	289 (97.0)		
Trace	32 (2.2)	27 (2.3)	5 (1.7)		
1+	19 (1.3)	17 (1.4)	2 (0.7)		
2+	5 (0.3)	4 (0.3)	1 (0.3)		
Missing	8 (0.5)	7 (0.6)	1 (0.3)		
Urine occult blood, n (%)				0.508	0.146
Negative	1093 (74.1)	865 (73.5)	228 (76.5)		
Trace	157 (10.6)	131 (11.1)	26 (8.7)		
1+	156 (10.6)	122 (10.4)	34 (11.4)		
2+	44 (3.0)	36 (3.1)	8 (2.7)		
3+	15 (1.0)	14 (1.2)	1 (0.3)		
Missing	10 (0.7)	9 (0.8)	1 (0.3)		

Compared to those without DR, patients with DR were younger (median 63.0 vs 67.2 years) and more often male (63.8% vs 47.1%). They also had poorer glycemic control—fasting glucose (6.2 vs 5.1 mmol/L) and HbA1c (6.5% vs 5.8%) were both higher—along with higher hemoglobin, serum creatinine, and lower HDL-C (all P < 0.05). Urine glucose positivity was strikingly more common in the DR group (55.8% vs 8.3%; **Supplementary Table S2**). These differences align with known risk factors and support the clinical significance of our model’s predictors.

### Model development and comparison

**Figure 2:**
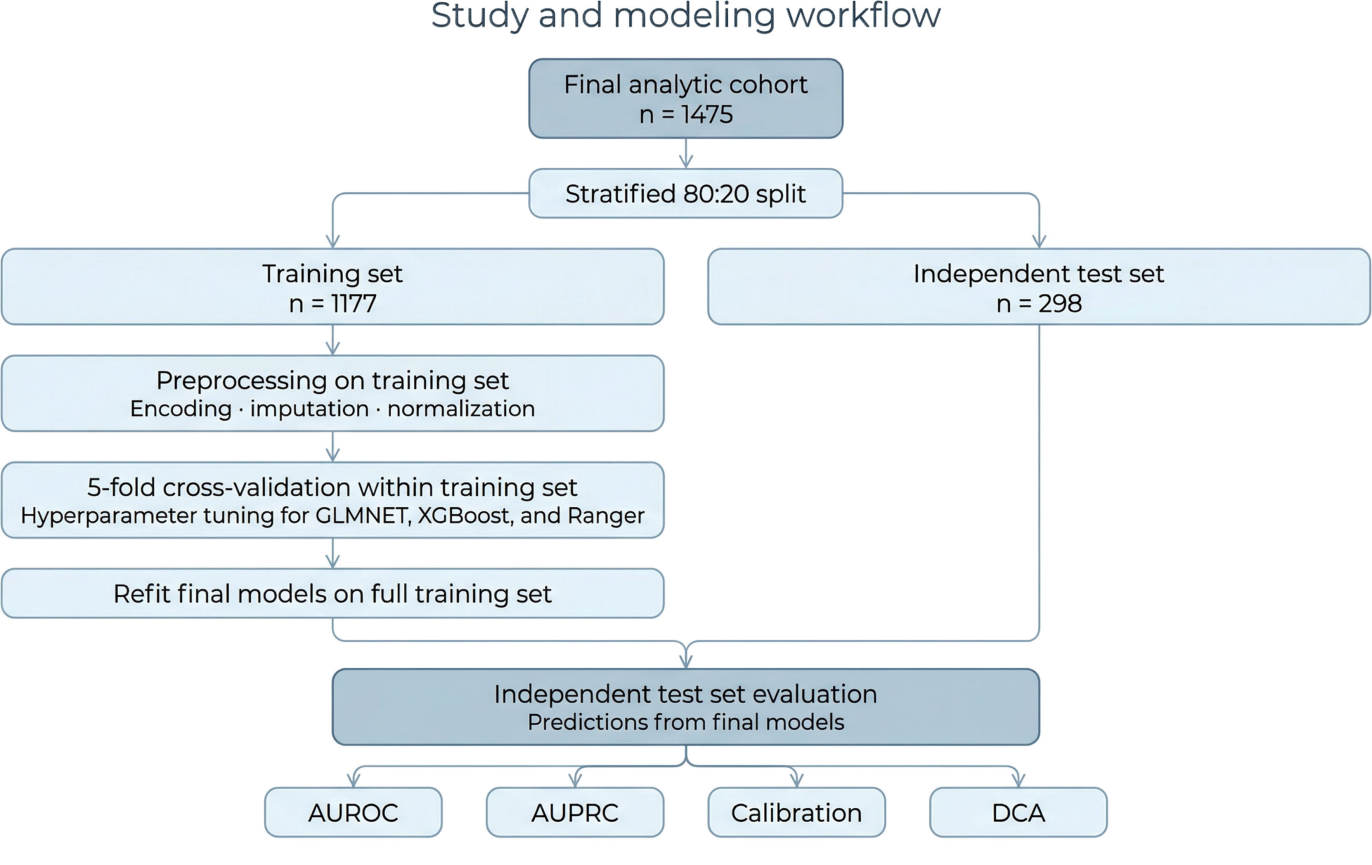
Study design and model development workflow. Schematic overview of data preprocessing, date split, model development, internal tuning, and independent test-set evaluation.

In the development set, we trained three candidate models using 5-fold cross-validation: penalized logistic regression (GLMNET), extreme gradient boosting (XGBoost), and random forest (Ranger) (**Figure 2**). When applied to the independent test set, GLMNET delivered the most balanced overall profile across discrimination, calibration, parsimony, and interpretability. XGBoost showed comparable discrimination, whereas Ranger performed less favorably. A detailed comparison of GLMNET, XGBoost, and Ranger in the held-out test set is provided in **Supplementary Table S3**. Based on its competitive predictive performance together with greater transparency and simpler structure, GLMNET was selected as the final model.

**Figure 3:**
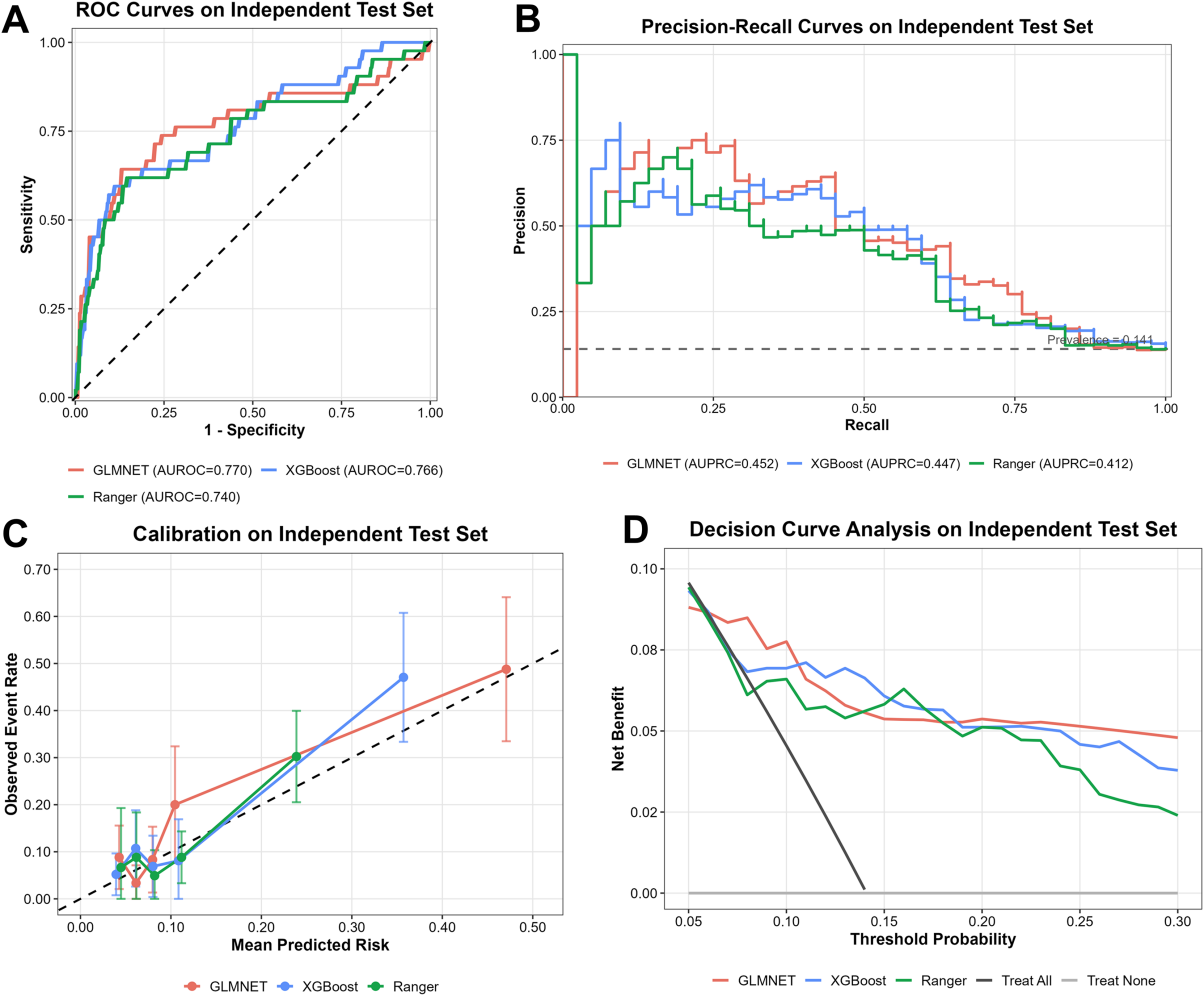
The performance of three machine learning models on the test set. **(A)** ROC curve showing discrimination performance of three machine learning models in the independent test cohort. **(B)** Precision-recall analyses comparing GLMNET, XGBoost, and Ranger for DR prediction. **(C)** Calibration of the three models. Calibration plots showing agreement between predicted and observed risk. **(D)** Clinical utility of the three models. Decision curve analysis showing the net clinical benefit across threshold probabilities.

The final GLMNET model yielded an AUROC of 0.770 (95% CI 0.671–0.856) in the test set (**Figure 3A**). Because DR prevalence was low (13.5%), we also examined the area under the precision-recall curve (AUPRC), which is more sensitive to performance in imbalanced settings. The AUPRC of 0.452 (95% CI 0.325–0.620) suggests acceptable precision given the disease frequency (**Figure 3B**). The Brier score was 0.095, indicating that predicted probabilities aligned reasonably well with observed outcomes. Calibration was acceptable, with an intercept of 0.206 and slope of 0.953 (**Figure 3C**; **Table 2**). On decision curve analysis, the model demonstrated positive net benefit across threshold probabilities from 10% to 40%, suggesting potential utility for risk stratification within this threshold range when ophthalmology resources are limited. Focused calibration and decision-curve visualizations for the final GLMNET model are provided in **Supplementary Figures S2** and **S3**. In sensitivity analyses, class weighting and random upsampling yielded slightly higher AUROC values but did not improve AUPRC and were associated with worse Brier scores (**Supplementary Figure S5**; **Supplementary Table S8**).

**Table 2:** Predictive performance of the candidate machine learning models in the independent test.

Model	N	Events	AUROC (95% CI)	AUPRC (95% CI)	Brier score	Calibration intercept	Calibration slope
GLMNET	298	42	0.770 (0.671–0.856)	0.452 (0.325–0.620)	0.095	0.206	0.953
XGBoost	298	42	0.766 (0.679–0.849)	0.447 (0.319–0.610)	0.099	0.296	1.026
Ranger	298	42	0.740 (0.645–0.830)	0.412 (0.290–0.567)	0.104	0.070	1.480

### Threshold-dependent performance

**Figure 4:**
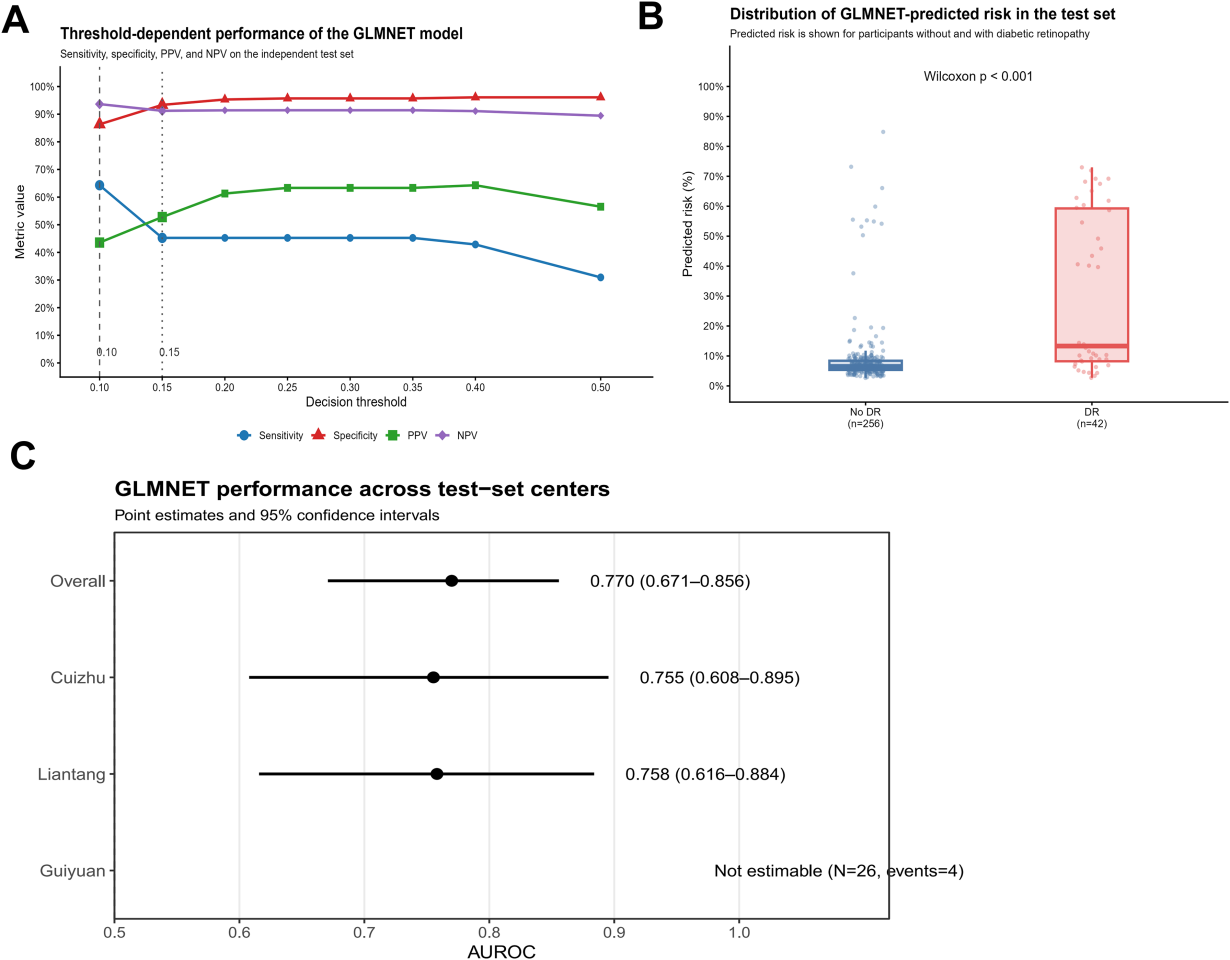
The performance of GLMNET model in test cohort. **(A)** Threshold-dependent performance visualization of the final GLMNET model in test set. Summary of sensitivity, specificity, predictive values, and related operating characteristics across a predefined range of probability thresholds. **(B)** Distribution of GLMNET model predicted risk by outcome group in test set. **(C)** Exploratory center-wise discrimination of the final GLMNET model. Center-stratified ROC curve of the final model within the multicenter cohort.

**Table 3** presented the model’s operating characteristics at selected risk thresholds. At a 10% threshold, sensitivity reached 64.3% and specificity 86.3%, with a negative predictive value of 93.6%. At a 20% threshold, specificity increased to 95.3% while sensitivity declined to 45.2%, and the positive predictive value rose to 61.3%. At a 40% threshold, specificity reached 96.1% with a PPV of 64.3% (**Supplementary Table S4**; **Figures 4A, B**). These results illustrated how the model can be calibrated for different clinical objectives: a lower threshold prioritized sensitivity for broad screening, whereas a higher threshold improved specificity for targeted referral when specialty resources were limited. A Youden-based cut-off was also examined but was presented as an exploratory reference rather than a fixed clinical recommendation.

**Table 3:** Threshold-based diagnostic performance of the final GLMNET model in the independent test cohort.

Threshold	Sensitivity	Specificity	PPV	NPV	Balanced accuracy
0.1	0.643	0.863	0.436	0.936	0.753
0.2	0.452	0.953	0.613	0.914	0.703
0.4	0.429	0.961	0.643	0.911	0.695

### Exploratory center evaluation

To explore whether model performance varied by site, we evaluated GLMNET separately in each of the three participating centers (**Supplementary Table S5**; **Figure 4C**). In the two larger centers—Cuizhu and Liantang, which together accounted for over 90% of the cohort—the model achieved broadly similar discrimination. The apparently higher AUROC observed in Guiyuan (0.898) should be interpreted cautiously because it was based on only 26 patients and 4 events. These findings suggest that model performance was not driven by any single site; however, this analysis should be interpreted as an exploratory between-center assessment rather than formal external validation, and validation in larger, more diverse populations is still required before drawing firm conclusions about transportability.

### Model interpretability analysis

**Figure 5:**
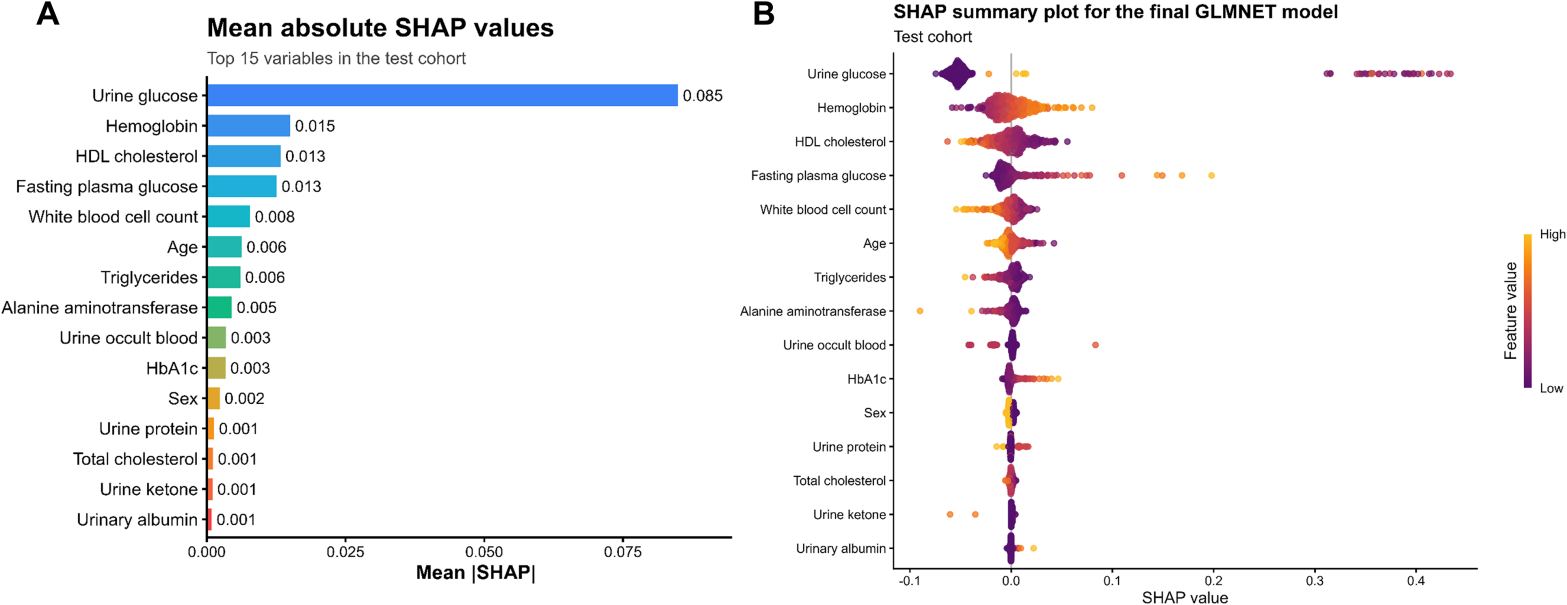
Feature importance analysis using SHapley Additive exPlanations (SHAP). **(A)** SHAP summary bar plot showing the SHAP values of each feature. **(B)** SHAP beeswarm plot displaying the distribution of SHAP values for each feature.

To understand how individual predictors contributed to the model’s risk estimates, we performed SHAP (SHapley Additive exPlanations) analysis on the final GLMNET model. **Figure 5** displays the SHAP summary plot, ranking predictors by their mean absolute SHAP values—a measure of global feature importance. Urine glucose emerged as the most influential predictor, followed by hemoglobin, HDL-C, fasting plasma glucose, and white blood cell count. Other factors also contributed, though with relatively lower importance. SHAP values showed higher values of urine glucose, hemoglobin, fasting plasma glucose were associated with increased DR risk, while higher HDL-C levels tended to lower predicted risk. These results showed how routine clinical data shaped the model’s risk estimates.

**Supplementary Table S6** presents the coefficients and odds ratios for predictors retained in the GLMNET model. Urine glucose showed the strongest association with DR, while higher HDL cholesterol (OR 0.41, P = 0.028) was protective. These findings align with the SHAP analysis, confirming urine glucose as the dominant risk factor and HDL as a protective factor. In a sensitivity analysis, refitting the final GLMNET model after excluding urine glucose resulted in lower AUROC and AUPRC and a worse Brier score (**Supplementary Table S7**), supporting that urine glucose contributed meaningful predictive information in this dataset, although its biological interpretation should remain cautious.

## Discussion

In this study, we developed and validated a clinical prediction model for diabetic retinopathy (DR) using routinely collected health data from three community-based health centers. Among the evaluated algorithms, penalized logistic regression (GLMNET) provided the most balanced overall profile in the independent test set. It achieved an AUROC of 0.77 with acceptable but imperfect calibration (intercept 0.21, slope 0.95). Given the low prevalence of DR, the AUPRC of 0.452 indicates moderate precision under class imbalance. On decision curve analysis, the model showed positive net benefit across the 10%–40% threshold range, suggesting possible utility for risk stratification in primary care where ophthalmic screening resources are limited, but not evidence of readiness for routine implementation.

The performance of our model (AUROC 0.77) falls within the range reported in the literature. A systematic review of DR prediction models reported AUROCs ranging from 0.70 to 0.96 across 15 studies, with most demonstrating moderate discriminative ability (11). Notably, all 15 studies were assessed as high risk of bias due to inadequate validation and poor reporting transparency. To address this, we conducted comprehensive evaluations of calibration and clinical utility. Another meta-analysis confirmed consistent predictors including diabetes duration, HbA1c, and renal function markers. However, most existing models were developed in hospital-based cohorts with higher disease prevalence (15). Our model’s performance, while more modest than some researches, may better reflect the realities of community-based primary care where early-stage disease predominates and prevalence is lower (13.5% in our cohort versus 25-32% in many hospital-based studies) (16). This distinction matters: models developed in tertiary settings often overestimate performance when applied to unselected primary care populations due to spectrum bias.

The final model retained routinely available clinical variables: age, glycemic and lipid indicators, hematological parameters, and urinalysis findings. These factors are consistent with established risk factors in other studies, increasing the credibility of our model (17, 18). They also offer insights into potential mechanistic pathways. The association between glycemic control and DR is well-established, reflecting the central role of chronic hyperglycemia in microvascular damage through advanced glycation end-product accumulation, oxidative stress, and inflammation. Lipid parameters, particularly triglycerides, may contribute through lipoprotein accumulation in retinal capillary endothelial cells and pericyte loss (19). Renal function markers likely reflect shared microvascular vulnerability, since diabetic nephropathy and retinopathy are both manifestations of systemic microvascular disease (20). The findings are consistent with other studies and suggest that routine clinical data, on its own, can capture physiologically relevant risk signals. Given its prominence in the model, urine glucose may function as a clinically accessible proxy for glycemic burden or broader metabolic dysregulation; however, its biological significance in relation to DR should not be overstated. The corresponding sensitivity analysis is shown in **Supplementary Table S7**.

None of the three models dramatically outperformed the others in discriminatory power, but GLMNET emerged as the most practical choice for the final model. The decision came down to a trade-off. XGBoost showed comparable performance in AUROC, but it exhibited poorer calibration and its black-box nature limits clinical transparency. This tension between predictive performance and interpretability is increasingly important to clinical adoption. Complex ensemble methods, despite great statistical metrics, often meet with resistance in real-world practice because clinicians need to understand the rationale behind a risk assessment before actually acting on it (21). Our finding that a relatively simple penalized logistic regression model performed competitively with more sophisticated algorithms echoes observations from other recent prediction modeling studies (22). For tools designed to support clinical judgment, interpretability is almost a necessity. This is particularly true in community settings, where physicians may have limited familiarity with black-box model outputs. GLMNET strikes an effective balance: it maintains reasonable predictive performance while offering the transparency and simplicity that facilitate real-world adoption.

Our findings have potential clinical relevance in resource-constrained primary care settings. Evidence from primary care settings consistently shows low adherence to DR screening recommendations—recent studies report non-adherence rates of 27–28% and one-year screening completion rates below 35% even with automated referral systems (23, 24). By identifying patients at relatively higher predicted risk, the model may help support more efficient allocation of limited ophthalmology resources. At a 20% risk threshold, specificity was high, reducing false-positive referrals, whereas a 10% threshold provided greater sensitivity and may support broader identification of higher-risk patients for proactive monitoring. The most appropriate threshold would depend on local capacity, referral pathways, and tolerance for false-positive and false-negative classifications. Importantly, the model relies mainly on routine data collected during diabetes management, such as demographics, laboratory tests, and vital signs, rather than retinal imaging. This may expand applicability to community and primary care settings where ophthalmologists and fundus cameras are usually scarce. Such a tool may help clinicians identify higher-risk patients for earlier referral or closer follow-up. In addition, unlike many black-box algorithms, the GLMNET model retains interpretability, which may improve clinical understanding of the factors driving risk estimates and could facilitate future implementation in real-world settings.

This study has several limitations. First, the data were derived from three urban community health centers and were not validated in a truly independent external cohort. Regional practice patterns, referral pathways, and case mix in these centers may differ from those in other healthcare systems or rural settings, which may limit model transportability. Second, the relatively low number of DR events affected the stability of AUPRC estimates and threshold-dependent performance, particularly for the smallest center. Third, the retrospective cross-sectional design introduces the possibility of information bias and does not capture the longitudinal progression of DR. Fourth, the analytic dataset contained a binary DR outcome only and did not permit severity-stratified modeling. Accordingly, this model should be regarded as a risk-stratification tool to support prioritization of screening or follow-up, rather than as a replacement for ophthalmic examination or specialist diagnosis. External validation and prospective implementation studies are needed before routine clinical use.

## Conclusion

We developed a diabetic retinopathy prediction model using routine clinical data from three community health centers. Among the evaluated algorithms, GLMNET provided competitive discrimination and calibration together with greater transparency than more complex models. These findings support its potential role as a risk-stratification tool in community-based care, but external validation and prospective implementation studies are required before routine clinical use.

## Data Availability

The raw data supporting the conclusions of this article will be made available by the authors, without undue reservation.

## References

[1] Meng Y Liu Y Ma Y Chen Z Duan R Jiang L . Global, regional, and national burden of blindness due to diabetic retinopathy, 1990-2021. Ophthalmol Ther. (2025) 14:2599–615. doi: 10.1007/s40123-025-01230-y. PMID: 40864420 PMC12413353

[2] Alobaid T Karalliedde J O'Connell MD Gnudi L Sheehan K Lim KK . The prevalence and progression of microvascular complications and the interaction with ethnicity and socioeconomic status in people with type 2 diabetes: a systematic review and meta-analysis. J Diabetes Res. (2025) 2025:3307594. doi: 10.1155/jdr/3307594. PMID: 39831033 PMC11742076

[3] Owens DR Gurudas S Sivaprasad S Zaidi F Tapp R Kazantzis D . Idf diabetes atlas: a worldwide review of studies utilizing retinal photography to screen for diabetic retinopathy from 2017 to 2024 inclusive. Diabetes Res Clin Pract. (2025) 226:112346. doi: 10.1016/j.diabres.2025.112346. PMID: 40578519

[4] Wong TY Sun J Kawasaki R Ruamviboonsuk P Gupta N Lansingh VC . Guidelines on diabetic eye care: the international council of ophthalmology recommendations for screening, follow-up, referral, and treatment based on resource settings. Ophthalmology. (2018) 125:1608–22. doi: 10.1016/j.ophtha.2018.04.007. PMID: 29776671

[5] Chauhan A Vale L Kankaria A Gupta V Kaur G N N . Assessing adoption of human and ai-enabled diabetic retinopathy screening in primary healthcare settings: findings from a pragmatic trial. Arch Public Health = Arch Belges Sante Publique. (2025) 83:268. doi: 10.1186/s13690-025-01757-3. PMID: 41214732 PMC12599091

[6] Aspelund T Thornórisdóttir O Olafsdottir E Gudmundsdottir A Einarsdóttir AB Mehlsen J . Individual risk assessment and information technology to optimise screening frequency for diabetic retinopathy. Diabetologia. (2011) 54:2525–32. doi: 10.1007/s00125-011-2257-7. PMID: 21792613

[7] van der Heijden AA Nijpels G Badloe F Lovejoy HL Peelen LM Feenstra TL . Prediction models for development of retinopathy in people with type 2 diabetes: systematic review and external validation in a dutch primary care setting. Diabetologia. (2020) 63:1110–9. doi: 10.1007/s00125-020-05134-3. PMID: 32246157 PMC7228897

[8] Bantounou MA Nahar TAK Plascevic J Kumar N Nath M Myint PK . Drug exposure as a predictor in diabetic retinopathy risk prediction models-a systematic review and meta-analysis. Am J Ophthalmol. (2024) 268:29–44. doi: 10.1016/j.ajo.2024.07.012. PMID: 39033831

[9] Lian J So C Mcghee SM Thach T Lam CLK Fung CSC . To determine the risk-based screening interval for diabetic retinopathy: development and validation of risk algorithm from a retrospective cohort study. Diabetes Metab J. (2025) 49:286–97. doi: 10.4093/dmj.2024.0142. PMID: 39478439 PMC11960201

[10] Liang Y Zhang X Mei W Li Y Du Z Wang Y . Predicting vision-threatening diabetic retinopathy in patients with type 2 diabetes mellitus: systematic review, meta-analysis, and prospective validation study. J Glob Health. (2024) 14:4192. doi: 10.7189/jogh.14.04192. PMID: 39391902 PMC11467770

[11] Huang H Wu Y Ye H Li J Chen L Huang X . Risk prediction models for diabetic retinopathy: a systematic review. Front Endocrinol (Lausanne). (2025) 16:1556049. doi: 10.3389/fendo.2025.1556049. PMID: 40717809 PMC12291684

[12] Xuehao C Dejia W Xiaorong L . Integration of immunometabolic composite indices and machine learning for diabetic retinopathy risk stratification: insights from nhanes 2011 - 2020. Ophthalmol Sci. (2025) 5:100854. doi: 10.1016/j.xops.2025.100854. PMID: 40778361 PMC12329596

[13] Wang T Luo W Tu Y Chou Y Wu Y . Prospective validation of deep-learning algorithms for diabetic retinopathy screening: a systematic review and meta-analysis. Surv Ophthalmol. (2026) 71:827–46. doi: 10.1016/j.survophthal.2025.11.012. PMID: 41344407

[14] Wong TY Sabanayagam C . Strategies to tackle the global burden of diabetic retinopathy: from epidemiology to artificial intelligence. Ophthalmologica J Int D'ophtalmologie Int J Ophthalmol Z Fur Augenheilkunde. (2020) 243:9–20. doi: 10.1159/000502387. PMID: 31408872

[15] Saputro SA Pattanaprateep O Pattanateepapon A Karmacharya S Thakkinstian A . Prognostic models of diabetic microvascular complications: a systematic review and meta-analysis. Syst Rev. (2021) 10:288. doi: 10.1186/s13643-021-01841-z. PMID: 34724973 PMC8561867

[16] Olivieri C Salato M Campanella A Marolo P Parisi G Neri G . Comparison of diabetic retinopathy screening between hospital-based multidisciplinary and general practice-based settings: insights from a regional study in Italy. Acta Diabetol. (2025) 62:263–9. doi: 10.1007/s00592-024-02354-6. PMID: 39160371 PMC11861431

[17] Wu C Qin H Wei M Li A Zhu Q Guo J . Association between glycated hemoglobin variability and risk of diabetic kidney disease and diabetic retinopathy in diabetic patients: a systematic review and meta-analysis. Front Endocrinol (Lausanne). (2026) 17:1703190. doi: 10.3389/fendo.2026.1703190. PMID: 41694560 PMC12901347

[18] Alarbash AH Almutairi SN Alazmi HR Almutairi A Almutairi A . The contributions of glycated hemoglobin (hba1c), triglycerides, and hypertension to diabetic retinopathy: insights from a meta-analysis. Cureus. (2025) 17:e79066. doi: 10.7759/cureus.79066. PMID: 40109786 PMC11920856

[19] Busik JV . Lipid metabolism dysregulation in diabetic retinopathy. J Lipid Res. (2021) 62:100017. doi: 10.1194/jlr.TR120000981. PMID: 33581416 PMC7892987

[20] Tang S An X Sun W Zhang Y Yang C Kang X . Parallelism and non-parallelism in diabetic nephropathy and diabetic retinopathy. Front Endocrinol (Lausanne). (2024) 15:1336123. doi: 10.3389/fendo.2024.1336123. PMID: 38419958 PMC10899692

[21] van Royen FS Weerts HJP de Hond AAH Geersing G Rutten FH Moons KGM . In humble defense of unexplainable black box prediction models in healthcare. J Clin Epidemiol. (2026) 189:112013. doi: 10.1016/j.jclinepi.2025.112013. PMID: 41077324

[22] Austin PC Lee DS Wang B . The relative data hungriness of unpenalized and penalized logistic regression and ensemble-based machine learning methods: the case of calibration. Diagn Progn Res. (2024) 8:15. doi: 10.1186/s41512-024-00179-z. PMID: 39501360 PMC11539735

[23] Liu J Gibson E Ramchal S Shankar V Piggott K Sychev Y . Diabetic retinopathy screening with automated retinal image analysis in a primary care setting improves adherence to ophthalmic care. Ophthalmol Retina. (2021) 5:71–7. doi: 10.1016/j.oret.2020.06.016. PMID: 32562885 PMC8546907

[24] Fenwick EK Aravindhan A Tan NC Tang WE Ng LP Wong WT . Non-adherence to diabetes microvascular complications follow-up screening in the primary care population: predictors, associated barriers, and facilitators. Diabetes Res Clin Pract. (2025) 224:112193. doi: 10.1016/j.diabres.2025.112193. PMID: 40252775

