# Supplementary material for: A clinically interpretable machine learning model for early detection of diabetic retinopathy in multiple community health centers
Source: Front Endocrinol (Lausanne). 2026 May 4;17:1834629. doi: 10.3389/fendo.2026.1834629 (PMC13180576; doi:10.3389/fendo.2026.1834629)
Supplement: Supplementary file 1 [file DataSheet1.docx]

**Supplementary Figure S1. Final analytic cohort and predefined fixed train–test split.**

*Recommended file name: Fig S1*


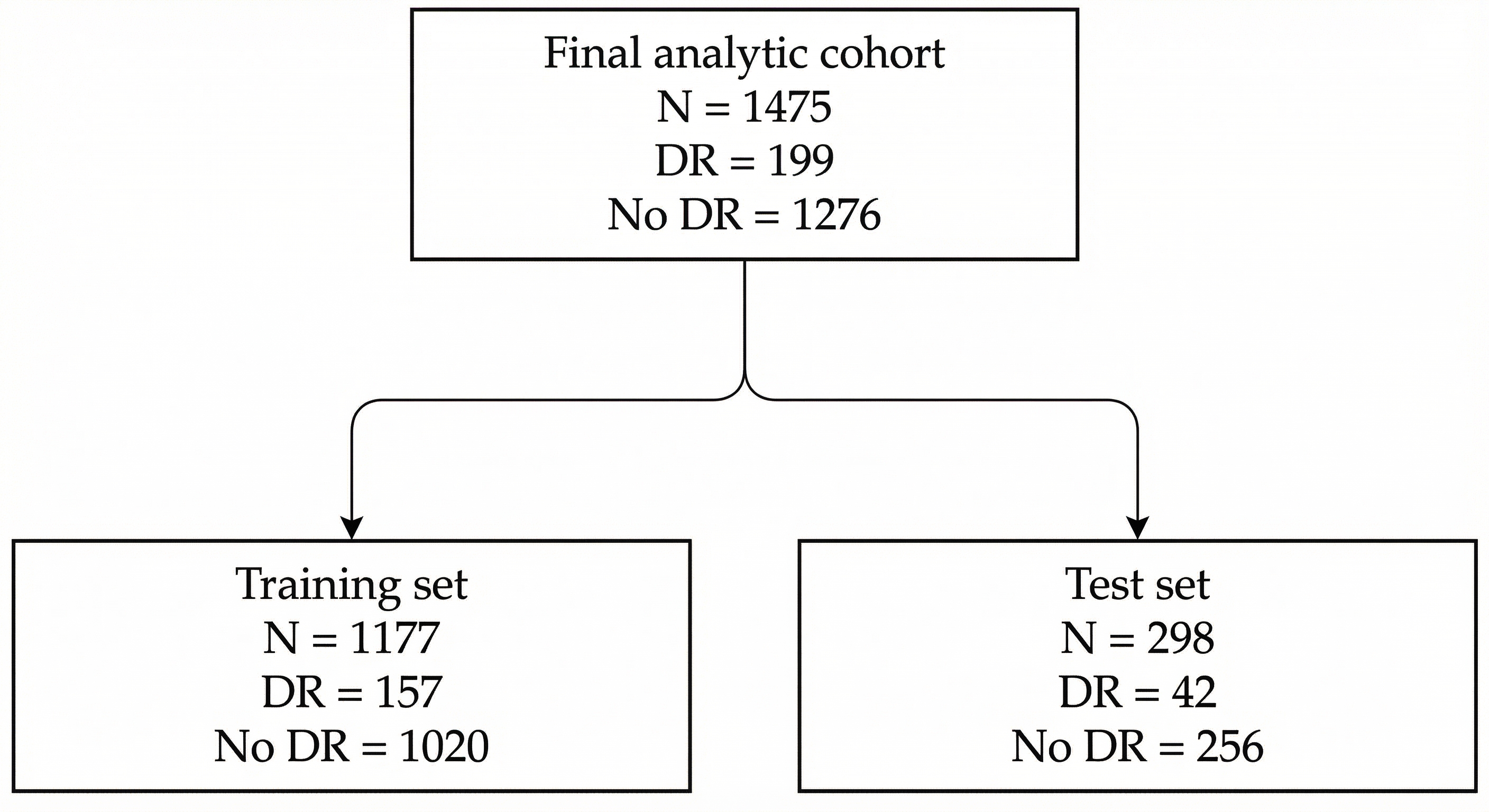


A total of 1,475 participants were included in the final analytic cohort, comprising 199 patients with diabetic retinopathy (DR) and 1,276 without DR. Under the predefined fixed data split, 1,177 participants (157 DR and 1,020 non-DR) were assigned to the development set and 298 participants (42 DR and 256 non-DR) to the held-out test set. This figure summarizes the final analytic cohort and the predefined train–test split used for model development and internal evaluation.

**Supplementary Figure S2. Calibration plot of the final GLMNET model in the held-out test set.**

*Recommended file name: Fig S2*


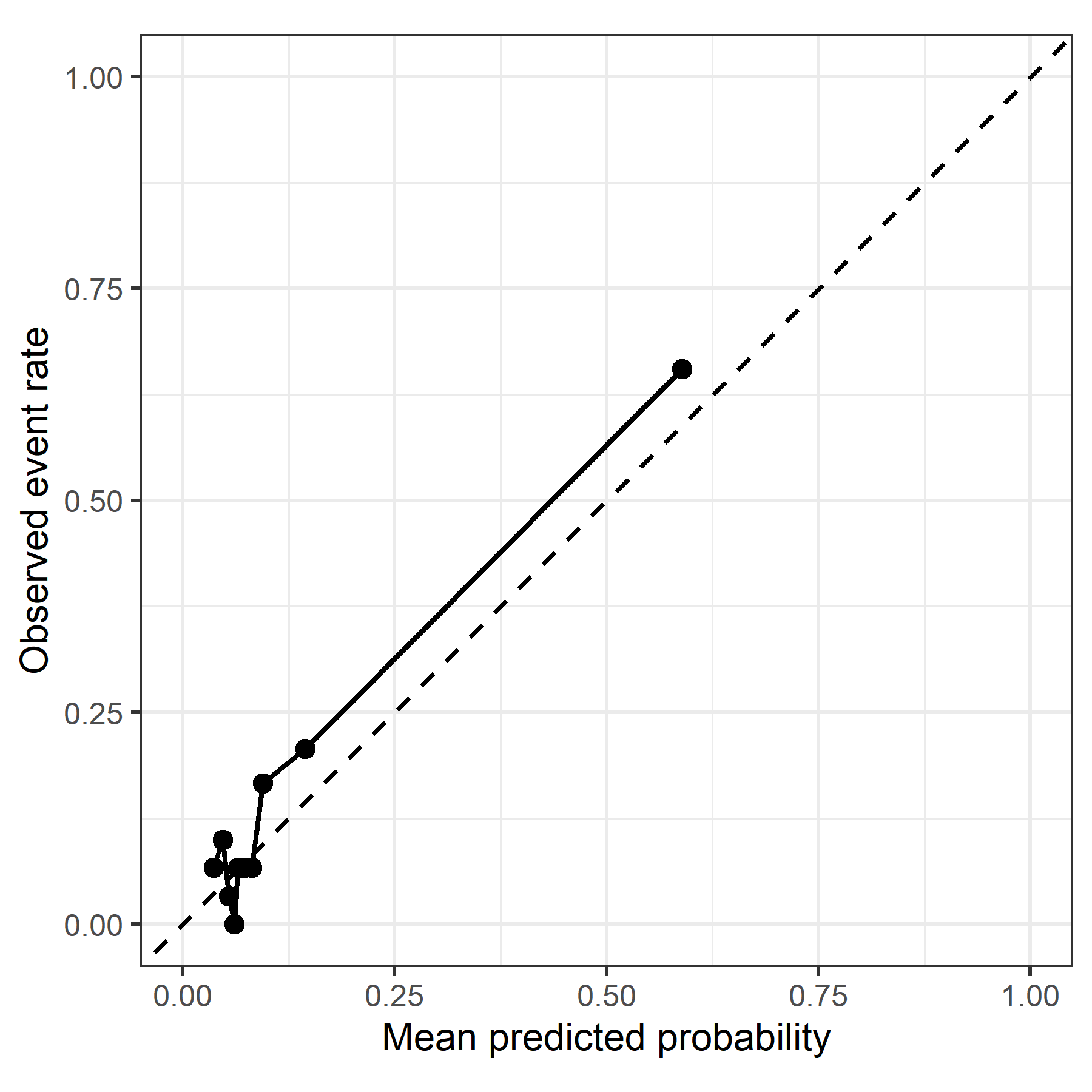


Calibration of the final GLMNET model was assessed in the held-out test set by comparing grouped observed event rates with mean predicted probabilities. The dashed diagonal line indicates ideal calibration. Overall, the model showed acceptable agreement between predicted and observed risk, with some underestimation of risk, consistent with a calibration intercept of 0.206 and a calibration slope of 0.953.

**Supplementary Figure S3. Decision curve analysis of the final GLMNET model in the held-out test set across the 10%–40% threshold range.**

*Recommended file name: Fig S3*


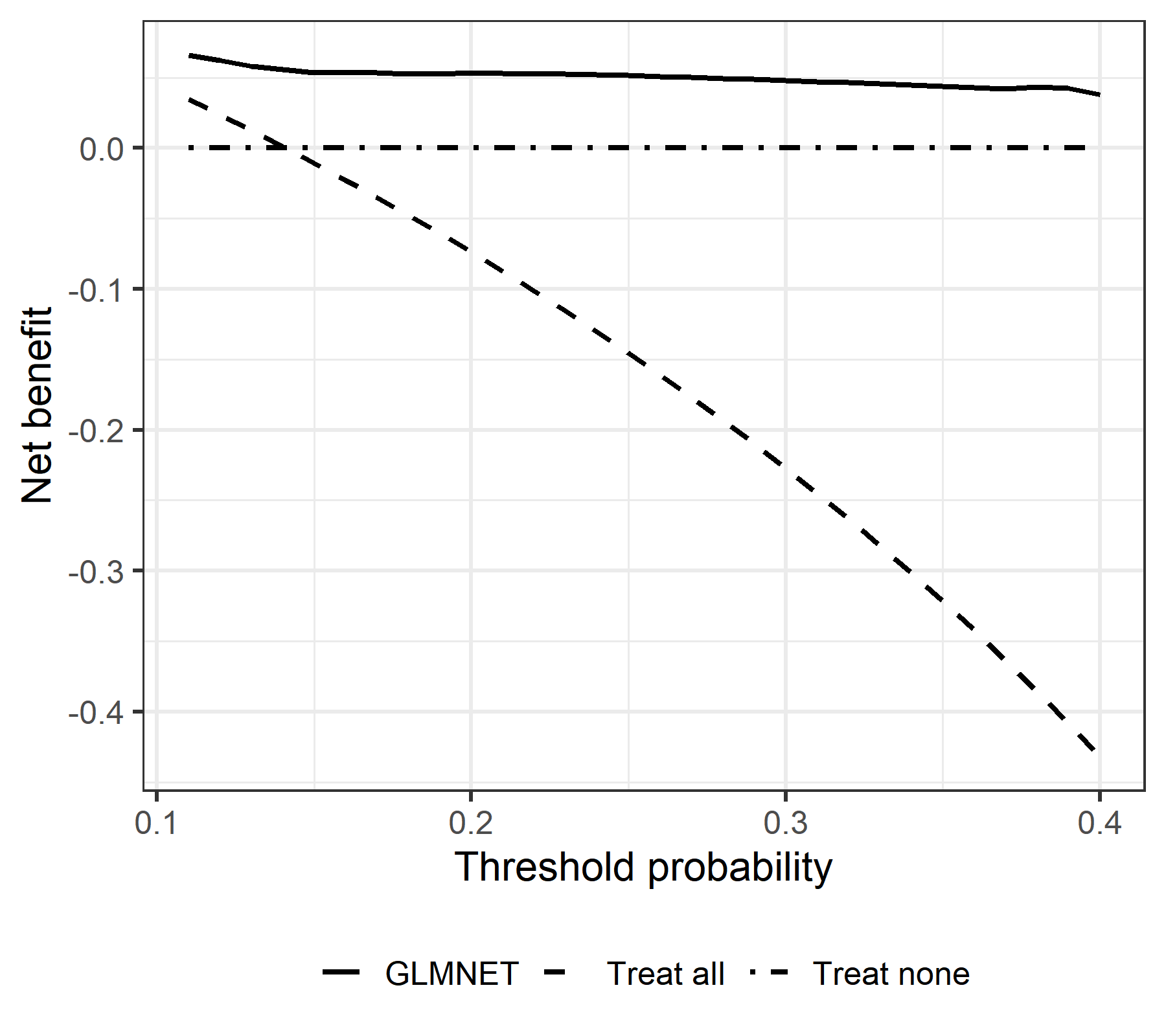


Decision curve analysis was performed for the final GLMNET model in the held-out test set. Net benefit was plotted against threshold probabilities from 10% to 40% and compared with the treat-all and treat-none strategies. Across this threshold range, the GLMNET model showed a higher net benefit than both default strategies, suggesting potential utility for risk-based referral or further ophthalmic assessment within this range.

**Supplementary Figure S4. Exploratory center-wise performance of the final GLMNET model on the held-out test set.**

Recommended file name: Fig S4


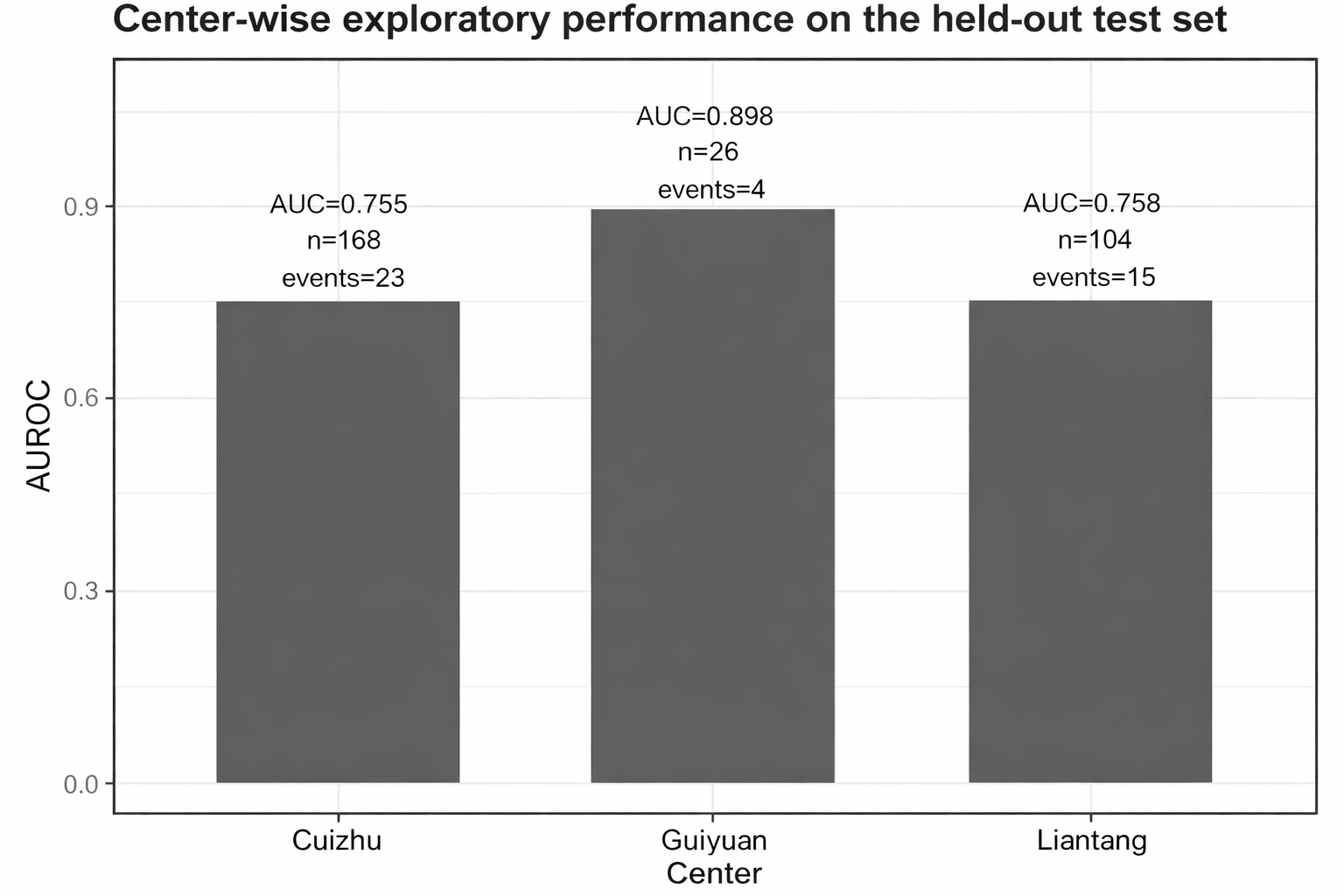


Exploratory center-wise AUROC of the final GLMNET model on the held-out test set. Performance was broadly comparable across the two larger centers, whereas the apparently higher AUROC in Guiyuan should be interpreted cautiously because it was based on only 26 patients and 4 events.

**Supplementary Figure S5. Sensitivity analysis of class-imbalance handling strategies.**

Recommended file name: Fig_S5


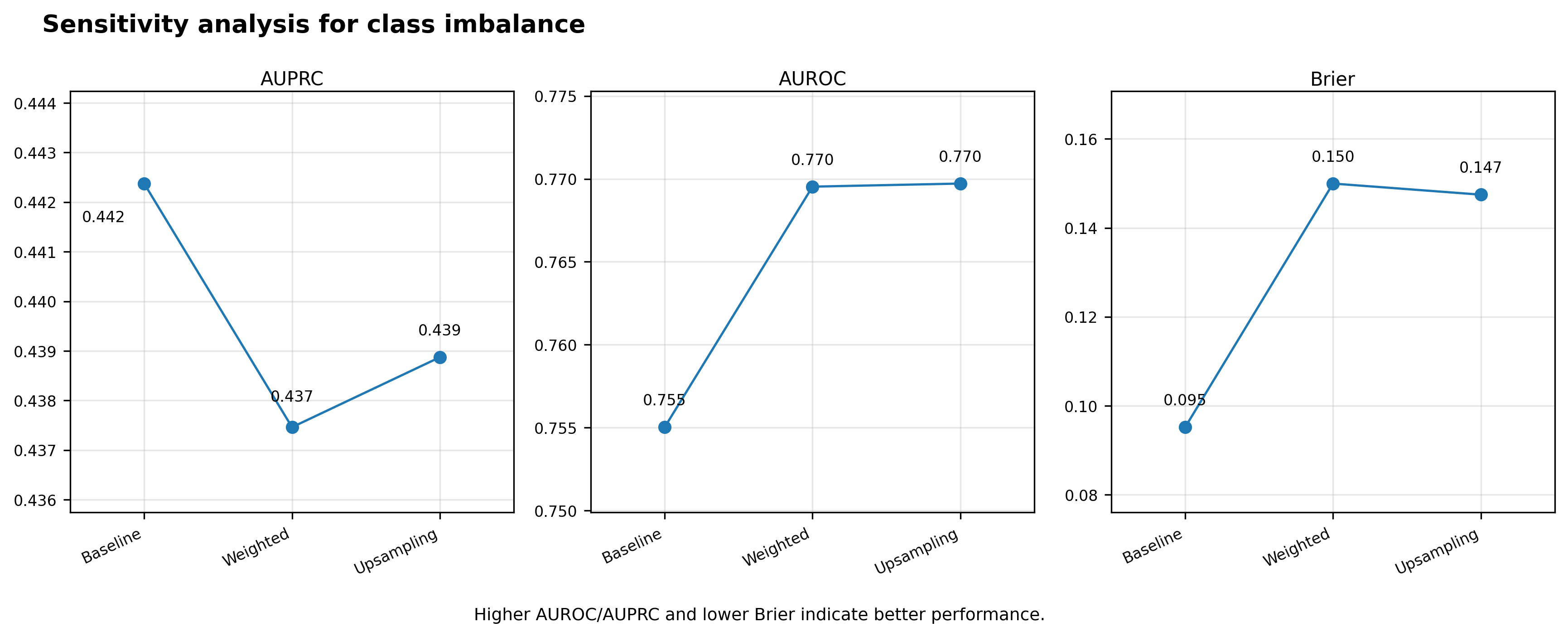


Comparison of the baseline GLMNET model with class-weighted and random upsampling GLMNET models using the fixed C-scheme train/test split. Model performance was evaluated by AUROC, AUPRC, and Brier score. Although the alternative imbalance-handling strategies yielded slightly higher AUROC values, they did not improve AUPRC and were associated with substantially worse Brier scores.
