# Supplementary material for: A clinically interpretable machine learning model for early detection of diabetic retinopathy in multiple community health centers
Source: Front Endocrinol (Lausanne). 2026 May 4;17:1834629. doi: 10.3389/fendo.2026.1834629 (PMC13180576; doi:10.3389/fendo.2026.1834629)
Supplement: Supplementary file 2 [file DataSheet2.docx]

**Supplementary Table S1. Variable-wise missingness in the overall cohort, development set, and held-out test set.**

This table summarizes the number and percentage of missing values for each variable before preprocessing and imputation.

| **Variable** | **n missing (Overall)** | **n missing (Train)** | **n missing (Test)** | **% missing (Overall)** | **% missing (Train)** | **% missing (Test)** |
| --- | --- | --- | --- | --- | --- | --- |
| IBbil, umolL | 108 | 94 | 14 | 7.32 | 7.99 | 4.70 |
| DBil, umolL | 107 | 93 | 14 | 7.25 | 7.90 | 4.70 |
| HbA1c | 55 | 42 | 13 | 3.73 | 3.57 | 4.36 |
| UAlb | 52 | 42 | 10 | 3.53 | 3.57 | 3.36 |
| PLT, 10^9^/L | 24 | 19 | 5 | 1.63 | 1.61 | 1.68 |
| urine_occult_blood | 10 | 9 | 1 | 0.68 | 0.76 | 0.34 |
| urine_protein | 8 | 7 | 1 | 0.54 | 0.59 | 0.34 |
| urine_glucose | 8 | 7 | 1 | 0.54 | 0.59 | 0.34 |
| urine_ketone | 8 | 7 | 1 | 0.54 | 0.59 | 0.34 |
| ALT, uL | 8 | 7 | 1 | 0.54 | 0.59 | 0.34 |
| AST, uL | 8 | 7 | 1 | 0.54 | 0.59 | 0.34 |
| Tbil_umolL | 8 | 7 | 1 | 0.54 | 0.59 | 0.34 |
| Scr, umolL | 6 | 5 | 1 | 0.41 | 0.42 | 0.34 |
| Bun, mmolL | 6 | 5 | 1 | 0.41 | 0.42 | 0.34 |
| TC, mmolL | 6 | 5 | 1 | 0.41 | 0.42 | 0.34 |
| TG, mmolL | 6 | 5 | 1 | 0.41 | 0.42 | 0.34 |
| LDL-C, mmolL | 6 | 5 | 1 | 0.41 | 0.42 | 0.34 |
| HDL-C, mmolL | 6 | 5 | 1 | 0.41 | 0.42 | 0.34 |
| Hb, g/L | 5 | 4 | 1 | 0.34 | 0.34 | 0.34 |
| Wbc, 10^9^/L | 5 | 4 | 1 | 0.34 | 0.34 | 0.34 |
| DBP | 4 | 3 | 1 | 0.27 | 0.25 | 0.34 |
| SBP | 4 | 3 | 1 | 0.27 | 0.25 | 0.34 |
| Age | 0 | 0 | 0 | 0.00 | 0.00 | 0.00 |
| Sex | 0 | 0 | 0 | 0.00 | 0.00 | 0.00 |
| Weight, kg | 0 | 0 | 0 | 0.00 | 0.00 | 0.00 |
| Waist, cm | 0 | 0 | 0 | 0.00 | 0.00 | 0.00 |
| BMI | 0 | 0 | 0 | 0.00 | 0.00 | 0.00 |
| FPG, mmolL | 0 | 0 | 0 | 0.00 | 0.00 | 0.00 |

*Abbreviations: Overall, full analytic cohort; Train, development set; Test, held-out test set.*

**Supplementary Table S2. Baseline characteristics of the study population with and without diabetic retinopathy.**

| **Study population** | **Overall** | **No_DR** | **DR** | **P value** | **SMD** |
| --- | --- | --- | --- | --- | --- |
| N | 1475 | 1276 | 199 |  |  |
| Center, n (%) |  |  |  | 0.933 | 0.028 |
| Cuizhu | 837 (56.7) | 725 (56.8) | 112 (56.3) |  |  |
| Guiyuan | 126 (8.5) | 110 (8.6) | 16 (8.0) |  |  |
| Liantang | 512 (34.7) | 441 (34.6) | 71 (35.7) |  |  |
| **Demographic characteristics** | **Demographic characteristics** |  |  |  |  |
| Age, years | 66.84 (59.60, 71.72) | 67.16 (60.79, 71.91) | 62.97 (55.30, 69.74) | <0.001 | 0.286 |
| Sex, n (%) |  |  |  | <0.001 | 0.341 |
| Male | 728 (49.4) | 601 (47.1) | 127 (63.8) |  |  |
| Female | 747 (50.6) | 675 (52.9) | 72 (36.2) |  |  |
| Weight, kg | 63.80 (56.50, 72.40) | 63.30 (56.40, 71.93) | 66.10 (57.60, 73.50) | 0.038 | 0.149 |
| WC, cm | 85 (79, 91) | 84.00 (78.00, 91.00) | 87.00 (80.50, 93.00) | 0.006 | 0.206 |
| BMI, kg/m² | 24.46 (22.43, 26.79) | 24.48 (22.45, 26.77) | 24.36 (22.27, 26.94) | 0.809 | 0.002 |
| **Blood pressure** |  |  |  |  |  |
| SBP, mmHg | 127 (117, 139) | 128.00 (117.00, 139.00) | 126.00 (114.50, 138.50) | 0.187 | 0.101 |
| DBP, mmHg | 81(74, 87) | 81.00 (74.00, 88.00) | 80.00 (74.00, 87.00) | 0.602 | 0.044 |
| **Glycemic variables** |  |  |  |  |  |
| FPG, mmol/L | 5.2 (4.8, 6.1) | 5.10 (4.70, 5.90) | 6.20 (5.20, 7.70) | <0.001 | 0.683 |
| HbA1c, % | 5.9 (5.5, 6.4) | 5.80 (5.50, 6.20) | 6.50 (5.70, 7.88) | <0.001 | 0.688 |
| **Hematologic variables** |  |  |  |  |  |
| Hb, g/L | 139 (130, 150) | 138 (129, 149) | 145 (136, 156) | <0.001 | 0.467 |
| WBC, ×10⁹/L | 6.02 (5.06, 7.09) | 6.02 (5.07, 7.07) | 5.99 (5.02, 7.22) | 0.632 | 0.062 |
| PLT, ×10⁹/L | 226 (193, 265) | 227 (193, 265) | 224 (191, 261) | 0.381 | 0.038 |
| **Liver function** |  |  |  |  |  |
| ALT, U/L | 18 (14, 26) | 18 (13, 26) | 19 (15, 27) | 0.174 | 0.014 |
| AST, U/L | 20 (18, 24) | 21 (18, 24) | 20 (17, 24) | 0.156 | 0.109 |
| TBil, μmol/L | 12.4 (10.0, 15.5) | 12.5 (10.0, 15.5) | 12.2 (10.2, 15.8) | 0.996 | 0.000 |
| DBil, μmol/L | 4.0 (3.0, 5.3) | 4.00 (3.00, 5.30) | 4.00 (3.10, 5.10) | 0.661 | 0.038 |
| IBil, μmol/L | 8.4 (6.7, 10.4) | 8.4 (6.7, 10.4) | 8.1 (6.8, 10.3) | 0.757 | 0.023 |
| **Renal function** |  |  |  |  |  |
| Scr, μmol/L | 69 (59, 82) | 69 (58, 82) | 73 (62, 87) | 0.003 | 0.142 |
| BUN, mmol/L | 5.3 (4.4, 6.2) | 5.2 (4.4, 6.2) | 5.8 (4.9, 6.7) | <0.001 | 0.283 |
| UAlb, mg/L | 13.10 (6.70, 26.85) | 13.00 (6.60, 26.00) | 14.30 (8.43, 32.85) | 0.053 | 0.145 |
| **Lipid profile mmol/L** |  |  |  |  |  |
| TC | 4.73 (4.04, 5.47) | 4.74 (4.06, 5.48) | 4.66 (3.93, 5.38) | 0.091 | 0.139 |
| TG | 1.35 (0.98, 1.97) | 1.34 (0.97, 1.94) | 1.40 (1.05, 2.04) | 0.444 | 0.014 |
| LDL-C | 2.85 (2.20, 3.43) | 2.85 (2.23, 3.44) | 2.79 (2.03, 3.40) | 0.263 | 0.091 |
| HDL-C | 1.23 (1.04, 1.49) | 1.24 (1.04, 1.50) | 1.16 (1.00, 1.40) | 0.005 | 0.229 |
| **Urinalysis** |  |  |  |  |  |
| Urine protein, n (%) |  |  |  | 0.122 | 0.201 |
| Negative | 1202 (81.5) | 1052 (82.4) | 150 (75.4) |  |  |
| Trace | 123 (8.3) | 101 (7.9) | 22 (11.1) |  |  |
| 1+ | 94 (6.4) | 78 (6.1) | 16 (8.0) |  |  |
| 2+ | 31 (2.1) | 27 (2.1) | 4 (2.0) |  |  |
| 3+ | 17 (1.2) | 12 (0.9) | 5 (2.5) |  |  |
| Missing | 8 (0.5) | 6 (0.5) | 2 (1.0) |  |  |
| Urine glucose, n (%) |  |  |  | <0.001 | 1.039 |
| Negative | 1258 (85.3) | 1170 (91.7) | 88 (44.2) |  |  |
| Trace | 12 (0.8) | 10 (0.8) | 2 (1.0) |  |  |
| 1+ | 22 (1.5) | 18 (1.4) | 4 (2.0) |  |  |
| 2+ | 12 (0.8) | 5 (0.4) | 7 (3.5) |  |  |
| 3+ | 53 (3.6) | 20 (1.6) | 33 (16.6) |  |  |
| 4+ | 110 (7.5) | 47 (3.7) | 63 (31.7) |  |  |
| Missing | 8 (0.5) | 6 (0.5) | 2 (1.0) |  |  |
| Urine ketone, n (%) |  |  |  | <0.001 | 0.325 |
| Negative | 1411 (95.7) | 1235 (96.8) | 176 (88.4) |  |  |
| Trace | 32 (2.2) | 20 (1.6) | 12 (6.0) |  |  |
| 1+ | 19 (1.3) | 13 (1.0) | 6 (3.0) |  |  |
| 2+ | 5 (0.3) | 2 (0.2) | 3 (1.5) |  |  |
| Missing | 8 (0.5) | 6 (0.5) | 2 (1.0) |  |  |
| Urine occult blood, n (%) |  |  |  | 0.049 | 0.269 |
| Negative | 1093 (74.1) | 929 (72.8) | 164 (82.4) |  |  |
| Trace | 157 (10.6) | 145 (11.4) | 12 (6.0) |  |  |
| 1+ | 156 (10.6) | 142 (11.1) | 14 (7.0) |  |  |
| 2+ | 44 (3.0) | 40 (3.1) | 4 (2.0) |  |  |
| 3+ | 15 (1.0) | 12 (0.9) | 3 (1.5) |  |  |
| Missing | 10 (0.7) | 8 (0.6) | 2 (1.0) |  |  |

*Abbreviations: WC, waist circumference; BMI, body mass index; SBP, systolic blood pressure; DBP, diastolic blood pressure; FPG, fasting plasma glucose; Hb, hemoglobin; WBC, white blood cell count; PLT, platelet count; ALT, alanine aminotransferase; AST, aspartate aminotransferase; TBil, total bilirubin; DBil, direct bilirubin; IBil, indirect bilirubin; Scr, serum creatinine; BUN, blood urea nitrogen; UAlb, urinary albumin; TC, total cholesterol; TG, triglycerides; LDL-C, low-density lipoprotein cholesterol; HDL-C, high-density lipoprotein cholesterol; DR, diabetic retinopathy; SMD, standardized mean difference.*

**Supplementary Table S3. Comparison of the final GLMNET model with more complex machine learning models in the held-out test set.**

GLMNET was retained as the final model because it offered competitive predictive performance together with greater interpretability and simpler implementation.

| **Model** | **AUROC** | **AUPRC** | **Brier score** | **Complexity** |
| --- | --- | --- | --- | --- |
| GLMNET | 0.770 | 0.453 | 0.095 | Simple / interpretable |
| XGBoost | 0.766 | 0.447 | 0.099 | More complex |
| Ranger | 0.740 | 0.412 | 0.104 | More complex |

**Supplementary Table S4. Threshold-based performance of the final GLMNET model in the independent test set.**

| **Threshold** | **Sensitivity** | **Specificity** | **PPV** | **NPV** | **Accuracy** | **F1 score** | **Balanced accuracy** | **LR+** | **LR-** |
| --- | --- | --- | --- | --- | --- | --- | --- | --- | --- |
| 0.10 | 0.643 | 0.863 | 0.436 | 0.936 | 0.832 | 0.519 | 0.753 | 4.702 | 0.414 |
| 0.15 | 0.452 | 0.934 | 0.528 | 0.912 | 0.866 | 0.487 | 0.693 | 6.812 | 0.587 |
| 0.20 | 0.452 | 0.953 | 0.613 | 0.914 | 0.883 | 0.521 | 0.703 | 9.651 | 0.575 |
| 0.25–0.35* | 0.452 | 0.957 | 0.633 | 0.914 | 0.886 | 0.528 | 0.705 | 10.528 | 0.572 |
| 0.40 | 0.429 | 0.961 | 0.643 | 0.911 | 0.886 | 0.514 | 0.695 | 10.971 | 0.595 |
| 0.50 | 0.310 | 0.961 | 0.565 | 0.895 | 0.869 | 0.400 | 0.635 | 7.924 | 0.719 |

**Identical performance metrics were observed for thresholds 0.25, 0.30, and 0.35; results are presented collectively. LR+, positive likelihood ratio; LR-, negative likelihood ratio.*

**Supplementary Table S5. Center-stratified performance of the final GLMNET model on the held-out test set.**

This table is presented as an exploratory between-center assessment rather than formal external validation. Calibration intercept and slope for Guiyuan were not estimated because of the limited number of DR events.

| **Center** | **N** | **Events** | **Event rate** | **AUROC (95% CI)** | **AUPRC (95% CI)** | **Brier score** | **Calibration intercept** | **Calibration slope** |
| --- | --- | --- | --- | --- | --- | --- | --- | --- |
| Overall | 298 | 42 | 0.141 | 0.770 (0.671–0.856) | 0.452 (0.325–0.620) | 0.095 | 0.206 | 0.953 |
| Cuizhu | 168 | 23 | 0.137 | 0.755 (0.608–0.895) | 0.464 (0.306–0.719) | 0.088 | 0.154 | 1.022 |
| Liantang | 104 | 15 | 0.144 | 0.758 (0.616–0.884) | 0.430 (0.230–0.661) | 0.112 | 0.240 | 0.744 |
| Guiyuan | 26 | 4 | 0.154 | 0.898 | 0.699 | 0.075 | NE | NE |

*Abbreviations: AUROC, area under the receiver operating characteristic curve; AUPRC, area under the precision-recall curve; NE, not estimated due to insufficient events.*

**Supplementary Table S6. Coefficients and odds ratios for predictors of diabetic retinopathy in the GLMNET model.**

| **Predictor** | **β** | **SE** | **OR (95% CI)** | **P value** |
| --- | --- | --- | --- | --- |
| Urine glucose: 2+ vs negative | 2.829 | 0.751 | 16.935 (3.884, 73.847) | <0.001 |
| Urine glucose: 3+ vs negative | 2.774 | 0.369 | 16.015 (7.765, 33.029) | <0.001 |
| Urine glucose: 4+ vs negative | 2.415 | 0.293 | 11.185 (6.292, 19.881) | <0.001 |
| Hemoglobin (per 10 g/L) | 0.165 | 0.091 | 1.179 (0.985, 1.410) | 0.072 |
| Fasting plasma glucose (mmol/L) | 0.130 | 0.083 | 1.139 (0.969, 1.339) | 0.116 |
| HbA1c (%) | 0.028 | 0.123 | 1.028 (0.807, 1.309) | 0.823 |
| Total cholesterol (mmol/L) | -0.013 | 0.105 | 0.987 (0.803, 1.213) | 0.902 |
| Triglycerides (mmol/L) | -0.190 | 0.093 | 0.827 (0.690, 0.993) | 0.041 |
| HDL cholesterol (mmol/L) | -0.883 | 0.402 | 0.414 (0.188, 0.910) | 0.028 |

*Abbreviations: SE, standard error; OR, odds ratio; CI, confidence interval.*

**Supplementary Table S7. Sensitivity analysis of the final GLMNET model after excluding urine glucose from the predictor set.**

| **Model** | **AUROC** | **AUPRC** | **Brier score** | **ΔAUROC vs original** | **ΔAUPRC vs original** | **ΔBrier vs original** |
| --- | --- | --- | --- | --- | --- | --- |
| GLMNET (original) | 0.770 | 0.453 | 0.095 | 0.000 | 0.000 | 0.000 |
| GLMNET without urine glucose | 0.707 | 0.347 | 0.110 | -0.062 | -0.106 | 0.015 |

*Δ indicates the difference relative to the original GLMNET model.*

**Supplementary Table S8. Sensitivity analysis of class-imbalance handling strategies.**

| **Model** | **AUROC** | **AUPRC** | **Brier score** |
| --- | --- | --- | --- |
| Baseline GLMNET | 0.755 | 0.442 | 0.095 |
| Class-weighted GLMNET | 0.770 | 0.437 | 0.150 |
| Random upsampling GLMNET | 0.770 | 0.439 | 0.147 |

*Compared with the baseline GLMNET model, class weighting and random upsampling did not provide consistent overall improvement and were associated with worse Brier scores. Higher AUROC/AUPRC and lower Brier indicate better performance.*
